# Childhood Adversities and Salivary Cortisol Responses to the Trier Social Stress Test: A Systematic Review of Studies Using the Children Trauma Questionnaire (CTQ)

**DOI:** 10.3390/ijerph18010029

**Published:** 2020-12-23

**Authors:** Chuk Ling Julian Lai, Daryl Yu Heng Lee, Monique On Yee Leung

**Affiliations:** 1 Psychophysiology Laboratory, Department of Social and Behavioural Sciences, City University of Hong Kong, Hong Kong, China; yhdlee2-c@my.cityu.edu.hk (D.Y.H.L.); moyleung2-c@my.cityu.edu.hk (M.O.Y.L.); 2 Department of Experimental Psychology, Division of Psychology and Language Sciences, University College London, London WC1H 0AP, UK

**Keywords:** childhood adversities, Childhood Trauma Questionnaire (CTQ), cortisol response, Trier Social Stress Test (TSST)

## Abstract

Alteration in cortisol response to acute social stressors has been hypothesized to mediate childhood adversities (CA) and increased morbidity in adulthood. However, the evidence supporting an association between CA and cortisol response to social stressors is inconclusive. The present review addressed this issue by reviewing the literature on CA and cortisol response to acute social stressors, with a focus on studies with adolescents or adults, using the Childhood Trauma Questionnaire (CTQ) to assess CA, and examining salivary cortisol response to the Trier Social Stress Test (TSST). Systematic searches of relevant articles in PsycINFO, PubMed, Web of Science and ScienceDirect in February and March 2020 identified 12 articles including 1196 participants with mean ages ranging from 15.3 to 52.3 yrs. across studies. CTQ scores were significantly associated with cortisol response in 2 studies. In addition, the physical abuse and emotional neglect subscales were associated with cortisol response respectively in 2 separate studies. The lack of association between CA and cortisol response calls for more longitudinal studies, and the use of formal records of maltreatment or informant reports in future research to complement information collected by retrospective measures. In addition, increased attention to biological mechanisms other than that associated with the regulation of cortisol in explaining the connection between CA and psychiatry morbidity is warranted.

## 1. Introduction

Childhood adversities (CA), which refers to the abuse and neglect of children under the age of 18 in the physical, emotional, and/or sexual domain, has been shown to be a global phenomenon [1], and one of the important social environmental factors that may explain morbidity [2] and mortality [3] in adulthood. The important health policy implication of the CA-morbidity association has triggered a surge of studies aiming at unravelling the mediating neurobiological mechanisms (e.g., [4]).

There is now evidence from both animal and human studies showing that exposure to maltreatments early in life or to childhood adversities (CA) contributes to pathological stress responses in adulthood (e.g., [5]). Moreover, the altered stress responses, which are mediated by the hypothalamic-pituitary-adrenocortical axis (HPAA), are associated with chronic disease [6] and increased psychiatric morbidity in adulthood [7]. Because of the important role of the HPAA in mediating the stress response, studies examining the connection between CA and cortisol, the end product of the HPAA, have proliferated in the last two decades [6,8]. It is now widely appreciated that the effect of CA on cortisol reactivity during critical periods of development interacts with specific genotypes to program long-lasting changes in glucocorticoid signaling via epigenetic alteration of the sensitivity of the glucocorticoid receptor (GR) (e.g., [4,9]). Recent studies have demonstrated the relevance of methylation of the GR receptor gene (*NR3C1*) promoter [10] and single nucleotide polymorphisms (SNPs) of the *FKBP5* gene in determining the sensitivity of GRs (e.g., [11,12]). The resulting changes in the cortisol negative feedback loop may alter the stress response and contribute to psychiatric and non-psychiatric vulnerability in adulthood [13]. Although CA has been shown to be associated with an attenuated cortisol response to social stressors (e.g., [14,15]), evidence regarding the effect of the *FKBP5* rs1360780 in modulating the association between CA and cortisol response to acute stressors is mixed. Buchmann et al. reported a significant effect of the *FKBP5* rs1360780 [15], which was not replicated by Alexander et al. with a comparable sample of healthy individuals [14].

The relationship between CA and cortisol response to the Trier Social Stress Test (TSST, [16]) and other laboratory stressors has been examined in recent meta-analytic reviews (e.g., [17,18]). The former [17] focused exclusively on studies looking at CA and salivary cortisol response to acute stressors. However, the latter [18] reviewed studies examining diurnal cortisol outcomes in addition to responses to both acute social stressors and pharmacological challenges, and thus is not relevant to the present review. CA was reported to be associated with a blunted salivary cortisol response to social stressors with a moderate effect size of *g* = −0.39 across 29 studies in Bunea et al. [17]. However, the interpretability of this pooled effect size is undermined by a substantial heterogeneity, *I*^2^ = 86.83%, which is high according to Higgins et al. [19]. In an earlier systematic review of CA and cortisol reactivity to stressors among infants and young children, Hunter et al. have also raised concerns about the heterogeneity of the adversity studied and stressors used to examine cortisol reactivity [20]. The studies included in Bunea et al. were heterogeneous in terms of mean ages of participants (9 to 62.7 years) and nature of CA (maltreatment vs. other family adversities) [17], although the TSST or modified versions were used in the majority of studies. In addition, the measures used to operationalize CA also varied across studies. Other than the Childhood Trauma Questionnaire (CTQ, [21]), self-report scales such as Childhood Traumatic Events Scale, the Traumatic Experiences Checklist, the Parental Bonding Instrument, the Childhood Experience of Care and Abuse, the Childhood Experiences of Violence Questionnaire, and the Early Trauma Inventory were also used either alone or in combination with CTQ in a number of studies. In a small number of studies, interviews such as the Childhood Adversity Interview and the Life History Calendar Interview were adopted. Although the effect size of blunted cortisol response was larger in studies using self-report retrospective questionnaires than those using either interviews or official records to measure CA, the heterogeneity potentially attributable to the use of the CTQ versus other self-report measures was not examined. In addition, the effect size of blunted cortisol response was also larger in studies with adults than those with either adolescents or children, and also in studies with a larger proportion of female participants. The substantial heterogeneity calls into question the suitability of the dataset used by Bunea et al. [17] for meta-analysis [17,22]. Although a number of moderators have been identified, the lack of substantive explanations for the effects of these moderators further curtails the impact of the meta-analysis. This suggests that useful information is more likely to be generated by limiting the heterogeneity of evidence to be reviewed, with a focus on “methodological subgroups” [19]: studies with specific target groups and/or using similar approaches in operationalizing CA.

Keeping the aforementioned issues in mind, we conducted the present review to address the following questions: (1) whether a blunted cortisol response to the TSST is consistently associated with higher CA in studies using the Childhood Trauma Questionnaire (CTQ), and (2) whether the total CTQ score or scores of subscales are more consistently associated with a blunted cortisol response. To sharpen the focus of our review, we examined studies (a) with participants who were 14 years or older, (b) using the TSST or an adapted version to induce social stress, and (c) operationalizing CA with the CTQ. Limiting the heterogeneity of evidence to be examined, we expected our review to arrive at conclusions more capable of informing future research. The reasons for focusing on the CTQ are the scale’s superior psychometric profile and prevalence in the literature. Although as many as nine retrospective measures of childhood adversities have been used in the literature, the CTQ is the only one that has been administered in a randomly selected sample [23]. In addition, the CTQ has been shown to be reliable (e.g., [24]), valid [25], and to exhibit a stable 5-factor structure across clinical, college and community samples (e.g., [21]). These five factors are Physical Neglect (PN), Physical Abuse (PA), Emotional Neglect (EN), Emotional Abuse (EA), and Sexual Abuse (SA). According to data from a large community adult sample [24], the Cronbach’s alpha was 0.91 for the whole scale, 0.58 for PN, 0.69 for PA, 0.85 for EN, 0.83 for EA and 0.94 for SA. The replicable 5-factor structure across clinical and healthy community samples has provided the major evidence for construct validity of the scale [21]. This demonstrates that the scale measures different types of maltreatment as it is designed to measure, and the contents of the five factors have similar meanings for different groups of people. Measurement invariance of the CTQ has also been demonstrated across gender and ethnicity by Thombs et al. [26]. With respect to criterion-related validity, Bernstein et al. have shown that therapists’ independent ratings on physical abuse, sexual abuse, emotional abuse and physical neglect were correlated significantly with patients’ scores on the corresponding factors of the CTQ [21] (p.185): 0.51 for PA, 0.75 for SA, 0.48 for EA, and 0.50 for PN.

With respect to prevalence, the CTQ can be considered as the most frequently used scale to assess CA in the literature. As illustrated in Table 1, the number of publications using CTQ in the last decade amounts to 2923, which is from 12 to 194 times higher than the number of publications using the other eight measures located by Burgermeister [23]. It is likely that research using self-report retrospective measures of CA has been dominated by the CTQ, which is in line with a recent global analysis of childhood maltreatment [1].

We focused on reactivity of salivary cortisol to the TSST instead of diurnal rhythm because early maltreatment has been shown to affect structures such as the hippocampus, amygdala and the prefrontal cortex that are sensitive to CA and relevant to the regulation of HPAA reactivity during acute stress [5]. Although these structures may also be involved in the regulation of circadian or diurnal rhythms of cortisol, the suprachiasmatic nucleus seems to play a more important role (e.g., [27]). Therefore, it is reasonable to expect that the effects of CA on cortisol are more likely to be observed in response to acute stressors rather than basal cortisol rhythms. This is in line with findings from a recent meta-analysis showing non-significant effects of CA on indices of the diurnal rhythm of cortisol [28]. Moreover, a recent longitudinal study also failed to demonstrate a significant association between CA and indices of diurnal salivary cortisol in a large Filipino sample [29]. The exclusive focus on TSST in our review is supported by the capability of this social stressor in provoking cortisol response consistently and effectively with a large effect size as reported in recent meta-analytic reviews (e.g., [30,31]).

Regarding the second question, the original CTQ consists of 70 items which was adapted to a 28-item short form by Bernstein et al. [21]. This short version was used in most studies subsequently and referred to as the CTQ instead of a short form of the original CTQ. The scale consists of 3 validity items and 25 clinical items measuring 5 aspects of maltreatment experiences: physical abuse (PA), physical neglect (PN), emotional abuse (EA), emotional neglect (EN) and sexual abuse (SA). Each item of the CTQ is rated on a 5-point scale with response options ranging from “never true” to “very often true”. As the 5 factors or subscales of the CTQ are only moderately correlated, the 5 factors can be scored separately to generate 5 subscale scores (e.g., [25]). On the other hand, because the 5 factors also load onto a higher order factor “Childhood Trauma” [24], the CTQ can also be scored as a 25-item scale to generate a CTQ total score. However, there is evidence showing that the factor structure of the CTQ differs between the two sexes in that PA is conceptually not applicable to females (e.g., [32]). Therefore, findings from studies with women and operationalize CA by CTQ total score should be interpreted with caution. In addition, specific subscale scores such as EN (e.g., [33]) or PN (e.g., [34]) have been shown to be more strongly associated with cortisol response to the TSST than the CTQ total score. This implies that the CTQ total score may not be as consistently associated with cortisol reactivity as are specific subscale scores. This issue has not been addressed by Bunea et al. and will thus be examined in the present review [17]. Due to the increasing use of CTQ in assessing CA, especially in studies with previously understudied populations (e.g., Chinese, [35]), demonstration of a consistent association between the CTQ or its subscales with cortisol reactivity would support the continual use of this scale in research on the health consequences of CA.

## 2. Method

**Figure 1:**
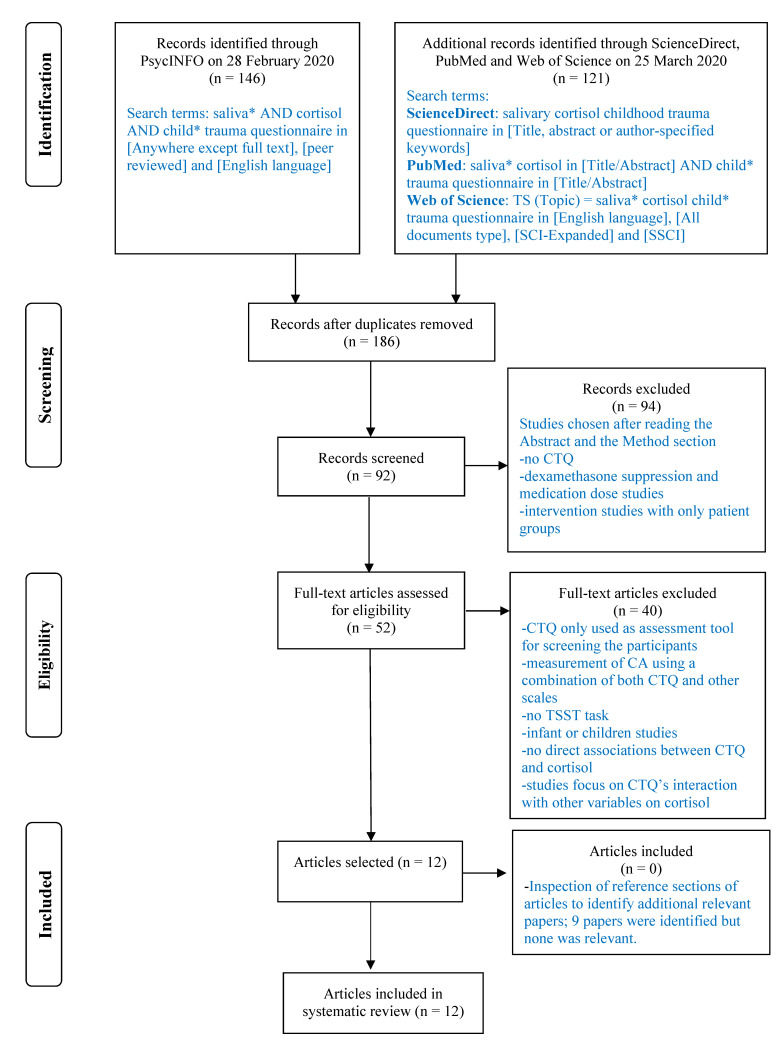
Study selection flow chart.

The present study was designed in accordance with the PRISMA guidelines for systematic reviews and meta-analyses [36]. We searched the databases PsycINFO, PubMed, Web of Science and ScienceDirect in February and March 2020, to locate eligible studies using specific strategies as illustrated in Figure 1. Articles were selected without any temporal restrictions (e.g., all dates) using the following inclusion criteria: (a) the CTQ, (b) TSST or modified versions, (c) salivary cortisol, (d) adolescent or adult participants, (e) written in English, and (f) published before February 2020. As this review focused on the association between the CTQ and salivary cortisol response to the TSST, studies that used the CTQ in combination with other measures of childhood maltreatment to derive a score or index of childhood maltreatment were excluded. In addition, studies that used the dexamethasone/CRH test to challenge the HPAA were also excluded because the focus of the present review was on cortisol response to social stressors.

## 3. Results

### 3.1. Search of Relevant Studies

We arrived at eligible studies through a systematic search in PsycINFO, PubMed, Web of Science and ScienceDirect using a combination of specific keywords as illustrated in Figure 1. A total of 186 articles were initially identified after removal of duplicates. These articles were then evaluated by examination of the abstract and the Method section. This excluded 94 articles which either did not use the CTQ to assess CA or used cortisol response to the dexamethasone/CRH test as the outcome measure. Among the remaining 92 articles, 52 had full text. These 52 articles were further screened by the authors to exclude studies that used the CTQ in combination with other measures of CA, did not use the TSST, did not provide sufficient information on CA and cortisol, or looked at infants or children. Forty studies were excluded and 12 were eligible for further analysis. The reference sections of these selected studies were searched for additional relevant publications. As no additional articles were thus identified, the 12 articles were included in this review. The full manuscripts of these 12 articles were then reviewed by the three authors for key information.

### 3.2. Summary of Included Studies

The 12 studies include 1196 participants and are grouped into 2 major categories of studies with healthy or clinical samples in Table 2. A brief description of each study is also provided. Healthy participants were examined in 4 studies [34,37,38,39] and the remaining 8 studies focused either on patients with specific psychiatric or medical conditions (n = 3, [33,40,41]) or both patients and healthy controls (n = 5, [42,43,44,45,46]). The 12 studies used either the standard TSST (n = 9) or an adapted version (n = 3) as the social stressor. In the standard TSST [16], participants were required to prepare a speech alone and then deliver the speech and complete a mental arithmetic task in front of an evaluating panel of judges. The typical speech is supposed to represent that for an interview for the participants’ ideal jobs. The child version of the TSST (TSST-C) used by Bick et al. and Cook et al. required participants to recite the story they wrote in front of a panel of judges instead of preparing and delivering a speech for a job interview [34,39]. In the TSST adapted for schizophrenics, participants were asked to talk about their physical appearance for the speech task [45]. The majority of the studies (n = 8) were with female participants exclusively. On the other hand, Table 3 summarizes information about the settings of the 12 studies and additional characteristics of the samples used in each study. The 12 studies were uniformly cross-sectional in design, with 5 conducted in the United States and the rest in different European countries including Germany, the Netherlands, Italy, Switzerland and Belgium. For the 4 studies that provided information on race or ethnicity of participants, 2 studies used a predominantly American-African youth sample [34,39] whereas the other two used a predominantly White/Caucasian adult sample [37,44]. In studies with psychiatric samples, the diagnoses were based on either the Diagnostic and Statistical Manual of Mental Disorders, Fourth Edition (DSM-IV) [42,43,44,45] or the Fifth Edition (DSM-5) criteria [40,46].

### 3.3. Main Findings

#### 3.3.1. Studies with Healthy Participants

The mean ages of participants in the four studies with healthy participants ranged from 15.3 years [34] to 30.4 years [38]. One study looked exclusively at females [38]. The standard TSST was used in two studies [37,38] whereas the child version of TSST (TSST-C) was used in the other two studies with adolescents [34,39]. To capture the change of cortisol over time before, during and after the TSST, saliva samples were collected seven times from before to 60 to 90 min after termination of the stressor in the four studies. Cortisol response or reactivity was operationalized either as the overall output of cortisol over the seven sampling time points or the increase in cortisol with reference to the level prior to the commencement of the stressor. CA was operationalized in three studies using a composite score of the cut-off of each of the 5 CTQ subscales to categorize participants into having no, mild-moderate, or moderate-severe trauma exposure [34,37,38]. Total CTQ scores were used by Cook et al. [39]. Information in Table 2 shows clearly that the CTQ composite scores or total CTQ scores were not significantly associated with cortisol response to the TSST in all four studies. The only significant association was between the physical abuse subscale PA and cortisol response in females reported by Carpenter et al. [38]. Females with PA exhibited a blunted cortisol response compared to their peers without exposure to PA. However, this finding should be interpreted cautiously because as pointed out earlier, the concept of PA has been shown not to be applicable to women [32]. In sum, there is no evidence showing an association between CA and cortisol response in healthy participants.

#### 3.3.2. Studies with Clinical Samples

The mean ages of participants in studies with clinical samples ranged from 24.6 [46] to 52.3 years [41]. Five studies compared clinical samples with healthy controls whereas the remaining three examined clinical samples exclusively. Seven of the eight studies looked exclusively at women and the sample sizes ranged from 30 to 113 [41,44]. All studies used the standard TSST except Lange et al. who adopted a TSST modified for patients with schizophrenia spectrum disorders (SSD) [45]. Saliva samples were collected from five to nine times from the beginning of the stressor to 60–90 min after termination of the stressor [41,47]. Similar to studies with healthy participants, cortisol response to TSST was also operationalized as the overall output and/or the change in cortisol level with reference to the level at the beginning of or prior to the stressor. CA was operationalized in two different ways across studies: (1) composite scores based on the specific cut-off of each of the 5 CTQ subscales and (2) the CTQ total score and/or specific subscale scores. A significant and negative association between CA and cortisol response was found in three studies with female patients. Aleknaviciute et al. studied one group of women borderline personality disorder (BPD) (n = 26) [42], another group with cluster C personality disorder (CPD) (n = 20), and one group of healthy controls (n = 35). The participants were divided into two groups of high vs. low CA using median split of CTQ total scores. The group with high CA was shown to have lower overall cortisol than the group with low CA. In another study with 40 female patients having chronic fatigue syndrome (CFS), Kempke et al. examined the associations between each CTQ subscales and the cortisol response to TSST [33]. Only EN was found to be negatively associated with the cortisol response in that higher EN was associated with a lower rise in cortisol from baseline to peak and also a lower AUC_i_. Dividing 41 female anorexic patients into two groups with and without CA using composites of cut-offs of the 5 subscales, Monteleone et al. [46] reported that patients with CA exhibited a lower AUC_i_ than patients without CA. However, the significant association observed in patients with personality disorders [42] was not replicated in two other studies with female patients diagnosed with personality disorders [43,44]. In a similar vein, the significant association between CA and cortisol response in patients with eating disorders reported by Monteleone et al. [46] was not observed in Monteleone et al. [40]. In sum, the evidence showing an association between higher CA and a blunted cortisol response is mixed. Moreover, there is no consistent evidence showing that the negative relationship between CA and cortisol response is more likely to be observed in patients with a specific psychiatric condition.

## 4. Discussion

The present review included 12 datasets in which CTQ scores were examined in relation to cortisol response to the TSST in a total of 1196 participants. The results show that the evidence demonstrating a consistent association between higher CA as measured by the CTQ or its subscales with a blunted cortisol response to TSST, is weak despite a stronger association in studies with clinical samples. With respect to the question of whether the CTQ subscale scores are more consistently associated with a blunted cortisol response than the CTQ total score, the evidence is also inconclusive. CTQ subscale scores were only examined in 3 of the 12 studies and only 2 of these looked both at the CTQ total score and the subscale scores. Although Carpenter et al. showed that only PA but not CTQ total score was associated negatively with cortisol response [38], this was the only one among the twelve studies showing such a pattern. The questionable applicability of the subscale of PA to females also weakens the credibility of this finding.

In spite of the preponderance of nonsignificant associations and inconsistency, one consistent finding did emerge from our review: the correlation between CA and cortisol response to TSST is uniformly negative in the four studies reporting a significant association. This is in line with findings from a 4-wave longitudinal study with adolescents whose CA was assessed by measures other than the CTQ [48]. In addition, all the four studies looked exclusively at female participants, with one at healthy participants [38] and the other three at psychiatric patients [33,42,46]. The percentage of female participants in a study has been reported to be positively associated with the strength of association between higher CA and lower cortisol response to social stressors in the meta-analytic review by Bunea et al. [17]. The reason for this is not immediately apparent although females have been found in a recent meta-analytic review to exhibit a lower cortisol response to TSST, particularly at the peak and recovery than their male peers [49]. CA could serve to further suppress the already lower cortisol response typical to females.

Another factor potentially modulating the association between CA and cortisol response is psychiatric morbidity. As mentioned earlier, three out of four studies reporting a significant association between higher CA and lower cortisol response were with female participants having either borderline personality disorders [42], chronic fatigue syndrome (CFS) [33], or eating disorders [46]. This could be attributed to the close association of alterations in the functioning of the HPAA with eating disorders (reviewed by Chami et al.) and borderline personality disorders (BPD) (reviewed by Drew et al.) [50,51]. Moreover, the link between CA and eating disorders [52] and BPD [53] has also been demonstrated in separate studies. Regarding the association between CA and CFS, there is evidence showing the mediating effect of altered HPAA functioning in this relationship (e.g., [54]). Taken together, recent evidence and findings of our study suggest that female gender and psychiatric morbidity may amplify the pathogenic effect of CA on cortisol response to social stressors, particularly the TSST. Our findings also suggest that resilience among individuals should not be overlooked because, in the absence of psychiatric morbidity, CA and altered cortisol response to stressors are less likely to be significantly correlated. This is in line with the finding that the correlation between CTQ scores and cortisol response to TSST was not significant in the four studies with healthy participants [34,37,38,39], even in youths from high-risk families [34,39]. Despite the importance of psychiatric morbidity, this factor was not examined in the meta-analysis conducted by Bunea et al. [17] because of the small number of studies, (n = 4) including groups with psychopathology. Findings of the present study may serve to fill this gap.

The impact of our findings is moderated by a number of limitations. Although CTQ is the most commonly used measure for childhood adversities, the agreement of this retrospective measure with prospective reports from informants is low [55]. This issue curtails the generality of our findings because individuals captured by these two different measures of CA are different. In addition, CTQ has been used for screening purposes and not been treated as a major predictor variable of cortisol responses in a number of studies. The exclusion of these studies reduced the number of studies included in our review. However, we believe that we have already included the most relevant articles. We did not impose any temporal restrictions on the search for relevant articles. The earliest article included in our review was published in 2011, Carpenter et al., which is also the earliest article on CTQ and cortisol response to TSST in the meta-analytic review by Bunea et al., [17,38]. Although five earlier articles published prior to 2011 [56,57,58,59,60] and two in 2011 [61,62] were included in the meta-analysis, these articles did not meet our inclusion criteria because CA was operationalized by other measures or methods (e.g., [56,57,58,59,61,62]), or by a combination of CTQ with other measures, to arrive at a composite CA score (e.g., [60]).

On the other hand, given the weak evidence supporting an association between CA and cortisol response to the TSST, future research should pay increased attention to biological mechanisms other than that associated with the HPAA in explaining the link between CA and psychopathology. Physiological adaptation to stress involves interactions among the HPAA, the autonomic nervous system and the immune system [63]. The secretion of proinflammatory markers from the immune system is a commonly observed response to stressors. There is a substantial amount of evidence demonstrating the association between CA and increased levels of proinflammatory markers (reviewed by Baumeister et al., 2016 [64]) as well as CA and morbidity (reviewed by Gill et al.) [65]. Given the relevance of inflammation to cardiometabolic and psychiatric disorders [66,67], the elevation in proinflammatory markers could be a plausible pathway leading CA to poor health in adulthood.

## 5. Conclusions

In conclusion, the evidence showing an association between CA as measured retrospectively by the CTQ and cortisol response to the TSST is weak, although this relationship is stronger in females with psychiatric conditions. This has several implications for future research. Despite the advantages associated with the use of retrospective measures to examine CA, the CTQ is not able to capture all individuals who might have been exposed to childhood maltreatment. Future research should complement retrospective measures by increasing the use of non-retrospective measures such as official records of maltreatment or informant reports. Given the weak association between CA and cortisol response to TSST, the mediating role of cortisol dysregulation in the link between CA and increased morbidity in adulthood could have been overstated in the literature. Focus of future studies should be adjusted to complement cortisol with other biomarkers such as proinflammatory cytokines that have been shown to be associated with CA and health outcomes. Moreover, the bulk of studies examining the relationship between CA and cortisol response to TSST are cross-sectional in design, which is inappropriate for testing the CA-cortisol response hypothesis because childhood adversities is supposed to be an antecedent of dysregulated cortisol responses to acute stressors in adolescence or adulthood. This calls for more longitudinal studies in future research.

## Figures and Tables

**Table 1:** Summary of Respective Counts of Different Instruments on PsycINFO.

Instrument	Year											Total Number
	2010	2011	2012	2013	2014	2015	2016	2017	2018	2019	2020	
Childhood Trauma Questionnaire	126	170	208	273	300	322	298	355	440	305	126	2923
Assessing Environments	2	1	3	4	1	0	3	1	0	0	0	15
Childhood Experience of Care and Abuse Questionnaire	4	5	5	9	10	9	8	10	11	13	8	92
Child Abuse and Trauma Scale	9	9	9	9	8	8	13	6	8	4	5	88
Stressful Life Events Screening Questionnaire	9	6	7	13	16	15	27	20	18	8	12	151
Comprehensive Child Maltreatment Scale	1	2	1	1	3	1	2	1	3	1	1	17
Early Trauma Inventory	13	19	25	14	22	30	29	27	29	14	8	230
Childhood Trauma Interview	3	8	8	7	11	6	4	4	3	0	2	56
Childhood Maltreatment Interview	1	4	2	2	2	3	1	3	1	0	1	20

*Note.* The search result was based on searching the name of each instrument as a phrase (i.e., enclosed with quotation marks) in the “Test & measure” field on *PsycINFO*, limited to English peer-reviewed journal articles. The search result was retrieved on 11 August 2020.

**Table 2:** Summary of 12 studies included in the review.

	N	Gender	Age (years)	CTQ Scoring	Stressor	Outcomes	*p* Value
**Healthy Samples**							
Alexander et al. (2018) [37]	200	50% females	18–30 Mean = 23.7	Composites of cut-offs of the 5 subscales	TSST	No association	*p* = 0.986 Z = 0.018
Bick et al. (2015) [34]	206	53% females	14.4–19 Mean = 15.3	Composites of cut-offs of the 5 subscales; subscale scores	TSST-C	-CTQ total scores had no association with cortisol response -no correlation between subscales and cortisol response	*p* = 0.877 Z = 0.155
Carpenter et al. (2011) [38]	110	100% females	18–61 Mean = 30.4	Composites of cut-offs of the 5 subscales; subscale scores	TSST	-No association for CTQ total scores -Significant association between higher PA and a blunted cortisol response	*p* = 0.087 Z = 1.711 *p* < 0.005 Z = 2.807
Cook et al. (2012) [39]	175	51.8% females	Mean = 15.4	Dichotomization of total CTQ scores into 2 groups of low vs. high in CA	TSST-C	No association between CA and cortisol response	r = 0.09 *p* = 0.236 Z = 1.175
**Clinical Samples**							
Aleknaviciute et al. (2016) [42]	81	100% females; BPD: n = 26 CPD: n = 20 HC: n = 35	Mean = 29.8	2 groups of high vs. low in CA: median split of total CTQ scores	TSST	-High CTQ group exhibited a lower cortisol response than the low CTQ group	*p* < 0.01 Z = 2.576
Duesenberg et al. (2019) [43]	98	100% females; BPD: n = 49 HC: n = 49	Mean = 30	CTQ total score	TSST	No correlation between CTQ total score and cortisol response	No information
Ehrenthal et al. (2018) [44]	113	100% females; BPD: n = 39 BPDS: n = 15 HC: n = 59	18–48 Mean = 25.9	CTQ total score	TSST	No association between CTQ total score and cortisol response	*p*s > 0.10 Z = 1.645
Kempke et al. (2015) [33]	40	100% female CFS patients	28–58 Mean = 41.9	CTQ subscales	TSST	EN was negatively related to cortisol response	*p*s < 0.05 Z = 1.96
Lange et al. (2017) [45]	50	32% females; SSD: n = 25 HC: n = 25	Mean = 39.7	CTQ total score	TSST *	No association between CTQ scores and cortisol response	*p*s > 0.05 Z = 1.645
Monteleone et al. (2018) [46]	41	100% females; Mal AN: n = 12 noMal AN = 12 HC: n = 17	Mean = 24.6	Composites of cut-offs of the 5 subscales	TSST	Mal AN group exhibited a lower cortisol response compared to the other 2 groups	*p* = 0.005 Z = 2.81
Monteleone et al. (2019) [40]	52	100% females with eating disorders; Anorexia: n = 29 Bulimia: n = 23	Mean = 25.5	CTQ total score and subscale scores	TSST	No association between CTQ total score or subscale scores and cortisol response	*p*s = 0.1 to 0.7 Z = 0.674
Tell et al. (2018) [41]	30	100% female patients with breast cancer	Mean = 52.3	CTQ total score	TSST	No association between CTQ and cortisol response	*p* = 0.33 Z = 0.974

*, an adapted version for schizophrenic patients. CTQ = Childhood Trauma Questionnaire, BPD = Borderline Personality Disorder, BPDS = Borderline Personality Disorder Symptoms, CFS = Chronic Fatigue Syndrome, CPD = Cluster C Personality Disorder, HC = Healthy Control, SSD = Schizophrenia Spectrum Disorders, Mal AN = Anorexics with childhood maltreatment, noMal AN = Anorexics with no childhood maltreatment; TSST = Trier Social Stress Test, TSST-C = TSST for children.

**Table 3:** Supplementary information for 12 studies included in the review.

Authors	Setting of Target Population	Population	Sample characteristics	Design
**Studies with healthy Samples**				
Alexander et al. (2018) [37]	Germany	General population	Caucasians; 98 adults with exposure to Childhood Adversities (CA) vs. 102 nonexposed controls	Cross-sectional
Bick et al. (2015) [34]	United States	High-risk youth from low-income families	83% American-African, 12% Caucasian, 5% Hispanic	Cross-sectional
Carpenter et al. (2011) [38]	United States	General population	Female adults; no information on race or ethnicity	Cross-sectional
Cook et al. (2012) [39]	United States	Youth from high-risk low income families	86.9% American-African	Cross-sectional
**Studies with clinical Samples**				
Aleknaviciute et al. (2016) [42]	The Netherlands	Clinical population	27 women with Borderline Personality Disorder (BPD), 20 women with cluster C Personality Disorder, 35 healthy female controls; diagnoses based on DSM-IV Axis II criteria; no information on race or ethnicity	Cross-sectional
Duesenberg et al. (2019) [43]	Germany	General and clinical population	49 women with BPD without comorbid psychiatric conditions and 49 healthy female controls free of psychiatric conditions; diagnoses based on DSM-IV Axis I and II criteria; no information on race or ethnicity	Cross-sectional
Ehrenthal et al. (2018) [44]	United States	Clinical population, community residents and university students	39 women with BPD, 15 women with BPD symptoms, 59 healthy female controls; diagnoses based on DSM-IV Axis I and II criteria; 77.9% White/Caucasian	Cross-sectional
Kempke et al. (2015) [33]	Belgium	Clinical population	40 female patients with Chronic Fatigue Syndrome (CFS); diagnoses based on Center for Disease Control and Prevention criteria for CFS; no information on race or ethnicity	Cross-sectional
Lange et al. (2017) [45]	Switzerland	Clinical and general population	25 patients with schizophrenia spectrum disorders (SSD) vs. 25 healthy controls; diagnoses based on DSM-IV criteria; no information on race or ethnicity	Cross-sectional
Monteleone et al. (2018) [46]	Italy	Clinical and general population	12 women with Anorexia Nervosa (AN) and CA exposure, 12 women with Anorexia Nervosa without CA exposure, 17 healthy female controls; diagnoses based on DSM-5 and the Structured Clinical Interview for DSM-5 Disorders–Research Version; no information on race or ethnicity	Cross-sectional
Monteleone et al. (2019) 40]	Italy	Clinical population	29 women with Anorexia Nervosa, 23 women with Bulimia Nervosa; diagnoses based on the Structured Clinical Interview for DSM-5 Disorders–Research Version; no information on race or ethnicity	Cross-sectional
Tell et al. (2018) 41]	United States	Clinical population	30 women with early stage breast cancer enrolling in a large intervention trial; no information on race or ethnicity	Cross-sectional
